# Bioleaching of tennantite concentrate: influence of microbial community and solution redox potential

**DOI:** 10.3389/fmicb.2023.1339549

**Published:** 2024-01-08

**Authors:** Shota Kondo, Kaito Hayashi, Idol Phann, Naoko Okibe

**Affiliations:** Department of Earth Resources Engineering, Faculty of Engineering, Kyushu University, Fukuoka, Japan

**Keywords:** tennantite, bioleaching, solution redox potential (Eh), microbial community, Fe oxidisers, activated carbon

## Abstract

Despite its growing importance as a Cu resource, studies on tennantite bioleaching are highly limited. One of the key challenges in processing such Cu-As sulfides is their refractoriness and the solubilisation of toxic As. The ultimate goal is to achieve selective bioleaching of Cu with simultaneous immobilisation of As in the leach residues. This study investigated the effectiveness of activated carbon (AC)-assisted bioleaching of tennantite concentrate using a mixed culture containing various “strong” and “weak” Fe-oxidising bacteria/archaea plus a S-oxidising bacterium, with particular emphasis on controlling the solution redox potential (Eh). In the initial flask bioleaching tests, a steady increase in Eh (up to 840 mV) was observed, reflecting the activity of “strong” Fe-oxidisers. In this situation, AC dosing effectively suppressed the Eh value and the highest Cu dissolution (70%) was obtained in the AC-0.01% system, while simultaneously immobilising As. In order to maximise Cu dissolution and As immobilisation, it was found preferable to target the Eh range of 650–700 mV during bioleaching. The next bioreactor tests used the mixed culture of the same origin, but had been subcultured a few generations further on tennantite concentrate. The Eh level remained unexpectedly low (~630 mV) for most of the leaching period, regardless of the AC dosage. It was later found that the bioreactor systems were almost exclusively dominated by *Sb. thermosulfidooxidans*, a “weak” Fe oxidiser with high Cu/As tolerance. In this case, there was no need to artificially suppress the Eh level by AC dosing and Cu leached readily to a final Cu dissolution of ~60% while As dissolution was suppressed to ~15%. Thus, depending on the microbial community that develops at the processing site, Eh control can be achieved either naturally by the activity of “weak” Fe-oxidisers as the predominant survivors under high Cu/As stress, or artificially by the addition of an Eh regulator such as a carbon catalyst.

## 1 Introduction

Copper is a key element in a wide range of industries, including automotive, electronics, alloys, and renewable energy. Although its consumption is growing significantly, especially in emerging markets, its supply faces challenges due to depleting reserves and declining grades. Copper-arsenic sulfide minerals such as enargite (Cu_3_AsS_4_) and tennantite (Cu_12_As_4_S_13_) are often found in association with other sulfide minerals in Cu deposits and can contribute to the overall Cu recovery (Filippou et al., 2007; Acevedo and Gentina, 2013). However, the presence of As can present challenges in metallurgical processes. Pyrometallurgy is not the most economically viable process for such As-bearing minerals, due to its intensive energy requirements and environmental measures to control and mitigate As emissions. Environmental regulations for As and other potentially hazardous elements are becoming increasingly stringent and compliance can add significant cost and complexity to pyrometallurgical processing (Long et al., 2012). On the other hand, hydrometallurgical processes are often preferred for As-bearing ores as they are generally less energy intensive and can leach and recover valuable metals while avoiding the release of As vapour, thus addressing the environmental and health concerns.

During the flotation process, As-rich concentrates can be produced as a waste if the ore being processed contains significant amounts of As-bearing minerals, while the other, cleaner Cu-rich concentrates are sent to the smelting circuit. To recover the residual Cu value from such flotation wastes, bioleaching, can be considered as one of the most promising technologies from an environmental and economic point of view (Stanković et al., 2015; Okibe et al., 2022). The conditions favoured by the hydrometallurgical reaction of Cu sulfides have been extensively studied, with more emphasis on chalcopyrite than on enargite. This discrepancy in research intensity is mainly due to the prevalence of chalcopyrite in Cu deposits. Research on the hydrometallurgy of enargite has been somewhat limited compared to chalcopyrite due to its lower abundance and the unique issues associated with its As content. Studies on tennantite are even more limited, due to its lower abundance and mineralogical complexity. Despite the lower occurrence of these As-bearing minerals (compared to chalcopyrite), it is essential to research and develop hydrometallurgical techniques for processing such minerals, particularly as ore grades decline and industry seeks to maximise the recovery of valuable metals while minimising environmental impact. The development of efficient and environmentally friendly methods for processing complex minerals such as tennantite remains an area of interest and importance in the field of hydrometallurgy.

In the case of chalcopyrite, it is widely recognised that the solution redox potential (Eh) values play a central role in determining the efficiency of Cu leaching. Different Eh ranges correspond to different states of chalcopyrite reactivity. Viramontes-Gamboa et al. (2007, 2010) suggested that chalcopyrite leaching with ferric sulfate is in its active state at <685 mV (SHE), which shifts to a bistable state at 685–755 mV, and then to a passive state at >755 mV with a strong passivation effect, resulting in reduced leaching efficiency. Hiroyoshi et al. (2008a,b) proposed a mechanism of chalcopyrite dissolution controlled by Eh values: At low Eh, chalcopyrite leaching is enhanced by Fe^2+^ and Cu^2+^, leading to the formation of intermediate chalcocite (Cu_2_S), which is then oxidised to yield Cu^2+^. This theory was also applicable to the bioleaching situation. Our previous studies have shown that chalcopyrite bioleaching can be maximised by using “weak” Fe-oxidisers, which naturally microbiologically control the Eh levels during the bioleaching reaction (Masaki et al., 2018). Where Eh naturally increases in the system due to the presence of “strong” Fe-oxidisers, the carbon catalyst can effectively suppress and control the Eh level in the bioleach liquors because the carbon catalyst surface acts as an electron mediator to couple the reduction of Fe^3+^ (Uchida et al., 2000) and the oxidation of reduced inorganic sulfur compounds (RISCs). The Eh level was thus controlled by counterbalancing microbial Fe^2+^ oxidation (Oyama et al., 2020, 2021;). The effect of activated carbon has also been reported in the bioleaching of low-grade primary Cu sulfide ores (Zhang and Gu, 2007).

In contrast to chalcopyrite, the dissolution of the mineral enargite is favoured under highly oxidising conditions (high Eh) (Lattanzi et al., 2008). Similarly, the dissolution of pyrite (FeS_2_) is also favoured under high Eh conditions. Therefore, one of the challenges in processing enargite-rich ores is that the dissolution of enargite at high Eh can be hindered by the presence of pyrite. This is due to the passivation of the enargite surface by Fe(III)-bearing minerals formed during the dissolution of pyrite. Our previous studies have shown that as a compromise to mitigate this competition effect, suppressing the Eh value to around <700 mV is preferable to maintain a more stable and prolonged Cu dissolution from enargite while minimising the passivation effect of Fe(III)-bearing minerals, allowing better control of the leaching process and Cu recovery (Oyama et al., 2018, 2020, 2021; Okibe et al., 2022). Accordingly, the need for Eh control is different for chalcopyrite and enargite. In the case of bioleaching enargite/chalcopyrite-bearing complex concentrates, the optimum Eh for maximum Cu dissolution increased with higher enargite/chalcopyrite ratios in the concentrate (Oyama et al., 2021).

One of the key challenges in the processing of enargite is the solubilisation of toxic As during leaching, and the ideal goal is to achieve selective bioleaching of Cu while simultaneously immobilising As in the leach residues. Some of the dissolved As(III) can be oxidised and subsequently immobilised during bioleaching (Escobar et al., 2000; Takatsugi et al., 2011; Tanaka et al., 2015). Bioleaching at elevated temperatures (typically in the range of 60–70°C) has several advantages, including more efficient oxidation and dissolution of enargite and precipitation of dissolved As as crystalline scorodite (Takatsugi et al., 2011; Okibe et al., 2014, 2017, 2020; Tanaka and Okibe, 2018). However, bioleaching at less energy-intensive, moderately thermophilic temperatures (e.g., 45°C) can also promote Cu dissolution and As immobilisation by controlling the Eh level during bioleaching reactions (Oyama et al., 2020; Okibe et al., 2022).

Our separate studies of biogenic scorodite formation have shown that amorphous precursors consisting of ferric arsenate (FeAsO_4_·nH_2_O) and basic ferric sulfate (MFe_x_(SO_4_)_y_(OH)_z_) are formed in the initial stage. Subsequently, these precursors are transformed into crystalline scorodite by the dissolution-recrystallization process, in which AsO_4_^3−^ ions compete and exchange with SO_4_^2−^ ions to precipitate with Fe^3+^ (Tanaka et al., 2018). A similar ferric arsenate precipitation mechanism was also likely to occur in the actual enargite bioleaching situation. This is because the loss of Eh control (leading to higher Eh) resulted in sudden pyrite dissolution, providing SO_4_^2−^ ions that compete with AsO_4_^3−^ and triggering the re-solubilisation of ferric arsenate precipitates (Oyama et al., 2021).

The similarities in the mineralogy of tennantite and enargite can present similar challenges. Tennantite is commonly found in hydrothermal sulfide deposits in various locations around the world (Zhou et al., 2023). In some ore deposits, tennantite can coexist with enargite, and its presence can influence the overall mineralogy of the deposit. However, the relative abundance of enargite is often much higher than that of tennantite, and therefore only limited studies are available on tennantite.

Therefore, this study investigated the effectiveness of carbon-assisted bioleaching (flask and bioreactor tests) on tennantite concentrate with the concept of Eh control with the dual objectives of (i) promoting steady Cu dissolution from tennantite while preventing the rapid dissolution of coexisting minerals such as pyrite, and (ii) minimising As solubilisation by stabilising ferric arsenate precipitates formed in the bioleach liquor.

## 2 Materials and methods

### 2.1 Minerals

**Figure 1:**
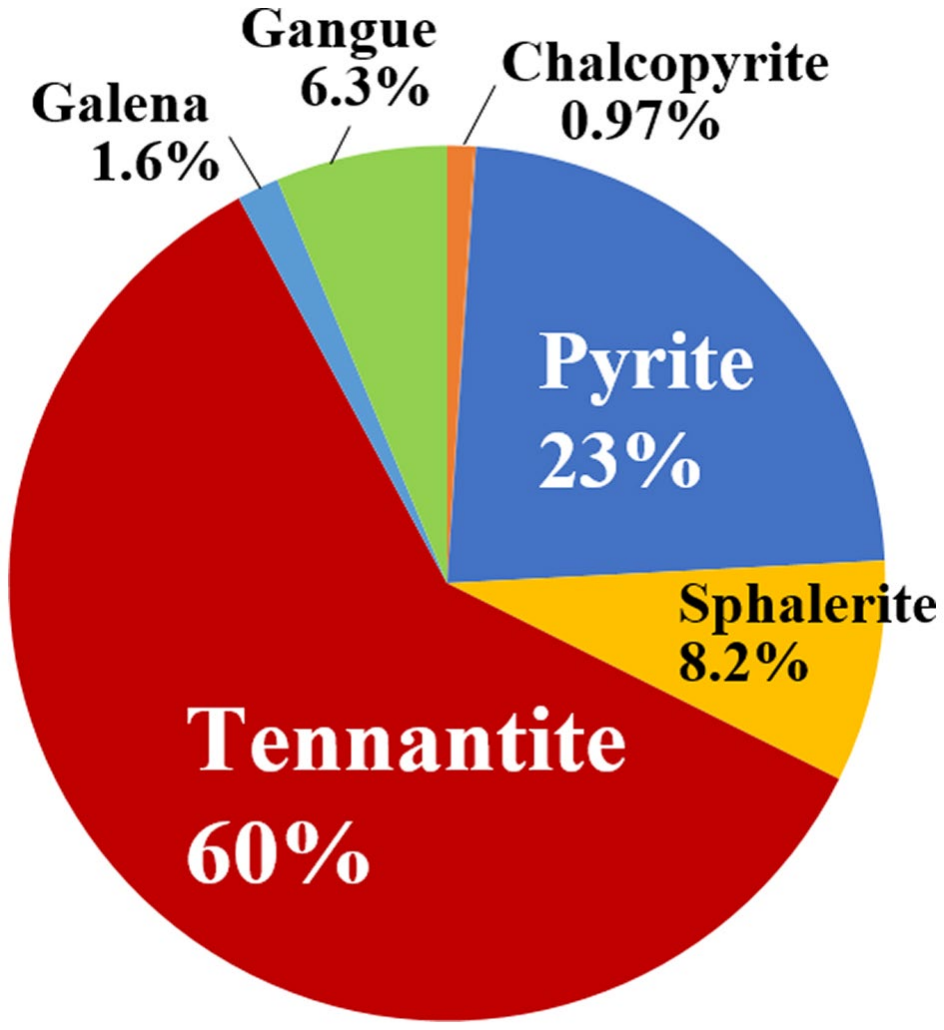
Mineral composition (wt %) of the tennantite concentrate used in this study, based on the scanning electron microscope (SEM)-based mineral liberation analysis (MLA).

A tennantite concentrate (D_50_ = 41 μm) consisting of 60% tennantite (Cu_12_As_4_S_13_), 23% pyrite (FeS_2_), 8.2% sphalerite (ZnS), 6.3% gangue, with minor amounts of chalcopyrite (CuFeS_2_) and galena (PbS) was used in this study (Figure 1). The elemental composition of the concentrate was as follows: S 31%, Cu 23%, Fe 12%, Zn 10%, Sb 7.8%, As 6.2%. The concentrate was used without washing or sterilisation.

### 2.2 Microorganisms

A mixed culture of six moderately thermophilic, acidophilic microorganisms was pre-grown on 5% tennantite, before being used as an inoculum for the flask bioleaching tests (as described in 2.3). The six acidophiles originally inoculated were: Fe-oxidising bacterium *Acidimicrobium* (*Am*.) *ferrooxidans* ICP (DSM 10331^T^), Fe-oxidising archaeon *Acidiplasma* sp. Fv-Ap, S-oxidising bacterium *Acidithiobacillus* (*At*.) *caldus* KU (DSM 8584^T^), and Fe/S-oxidising bacteria *Sulfobacillus* (*Sb*.) *sibiricus* N1 (DSM 17363^T^), *Sb. thermotolerans* Kr1 (DSM 17362^T^) and *Sb. thermosulfidooxidans* AT-1 (DSM 9293^T^). Subcultures were maintained aerobically at 45°C, shaken at 150 rpm in acidophile basal salts (ABS) medium (0.5 g/L MgSO_4_⋅7H_2_O, 0.45 g/L (NH_4_)_2_SO_4_, 0.15 g/L Na_2_SO_4_⋅10H_2_O, 0.05 g/L KCl, 0.05 g/L KH_2_PO_4_, 0.014 g/L Ca(NO_3_)_2_⋅4H_2_O; pH_ini_ 1.5 with H_2_SO_4_) containing 0.02% (w/v) yeast extract and 10 mM Fe^2+^. In our previous studies, the mixed culture of *Am. ferrooxidans* ICP, *At. caldus* KU and *Sb. sibiricus* N1 was shown to be effective for the bioleaching of As-containing minerals, thus included in this study (Tanaka et al., 2015; Okibe et al., 2022).

### 2.3 Bioleaching of tennantite concentrates (flask tests)

First, bioleaching tests were performed in Erlenmeyer flasks (500 mL) containing 200 mL ABS medium (pH_ini_ 1.5 with H_2_SO_4_) with 0.02% yeast extract, 5 mM Fe^2+^ and 5% (w/v) tennantite concentrate. Pre-grown mixed culture (section 2.2) was inoculated at 10% (v/v). Activated carbon (AC; CAS RN 7440-44-0, Wako; mean particle size 46.5 μm; specific surface area 1,400 m^2^/g) was added as catalyst to control the Eh value at different concentrations (0, 0.01, 0.025, 0.05% (w/v)). Flasks were incubated at 45°C, shaken at 150 rpm for 50 days. Liquid samples were taken periodically to monitor pH and E_h_ (vs. SHE). Liquid samples were filtered (0.45 μm) prior to the ICP-OES analysis (for total soluble concentrations of Fe, Cu, As, Zn and Sb) (Optima 8,300, Perkin Elmer; Rodgau, Germany). All tests were performed in duplicate flasks.

### 2.4 Bioleaching of tennantite concentrates (bioreactor tests)

Bioleaching tests were then performed in pH-controlled bioreactors (Bioneer-Neo 2.0 L, Marubishi Bioengineering; Tokyo, Japan) containing 1.2 L of ABS medium with 0.02% yeast extract and 5% tennantite concentrate. The pre-grown mixed culture from the flask bioleaching test (section 2.2) was inoculated at 10% (v/v). Two bioreactor systems (AC-free or AC 0.01%) were operated in parallel at 45°C, stirred at 150 rpm for 100 days. The pH was maintained at 2.0 from day 0 to day 37 and at 1.5 from day 38, by automatic addition of 0.5 M H_2_SO_4_. On day 53, 20 mM Fe^2+^ was added to the AC-free system and 10 mM Fe^2+^ was added to the AC-0.01% system. Liquid samples were taken periodically to monitor pH, E_h_ (vs. SHE), cell density (direct cell counting using a Thoma chamber) and concentrations of Fe^2+^, total Fe, total Cu, total As, total Zn and total Sb as described in section 2.3.

### 2.5 Solid and microbial community structure analyses

Bioleach residues were periodically collected from the bioreactors (on days 0, 40, 80 and 100) and freeze-dried overnight for X-ray diffraction (XRD; Rigaku; Ultima IV; CuKα 40 mA, 40 kV) analysis and scanning electron microscopy (AEM; VE-9800, KEYENCE) observation. The microbial community structure was analysed by extracting genomic DNA from bioleach residues (after the flask or bioreactor tests) (ISOIL for beads Beating; Nippon Gene; Toyama, Japan), subjected to 16S rDNA amplicon sequence analysis (V3-V4 region; MiSeq, Illumina) and compared with databases using Metagenome@KIN (TechnoSuruga Laboratory Co. Ltd.; Shizuoka, Japan).

### 2.6 As(III) tolerance test for *Sb. Thermosulfidooxidans*

*Sb. thermosulfidooxidans* was inoculated (at 1.0 × 10^7^ cells/ml) into 100 mL of ABS medium (pH 1.5) containing 0.02% yeast extract and different concentrations of As(III) (as NaAsO_2_; 0, 2.6, 6.5, 13 or 26 mM) in 300 mL flasks. The flasks were incubated shaken at 100 rpm, 45°C. Samples were taken periodically to monitor cell density (using a bacterial counting chamber). Experiments were conducted in duplicate flasks.

## 3 Results and discussion

### 3.1 Bioleaching of tennantite concentrates (flask tests)

**Figure 2:**
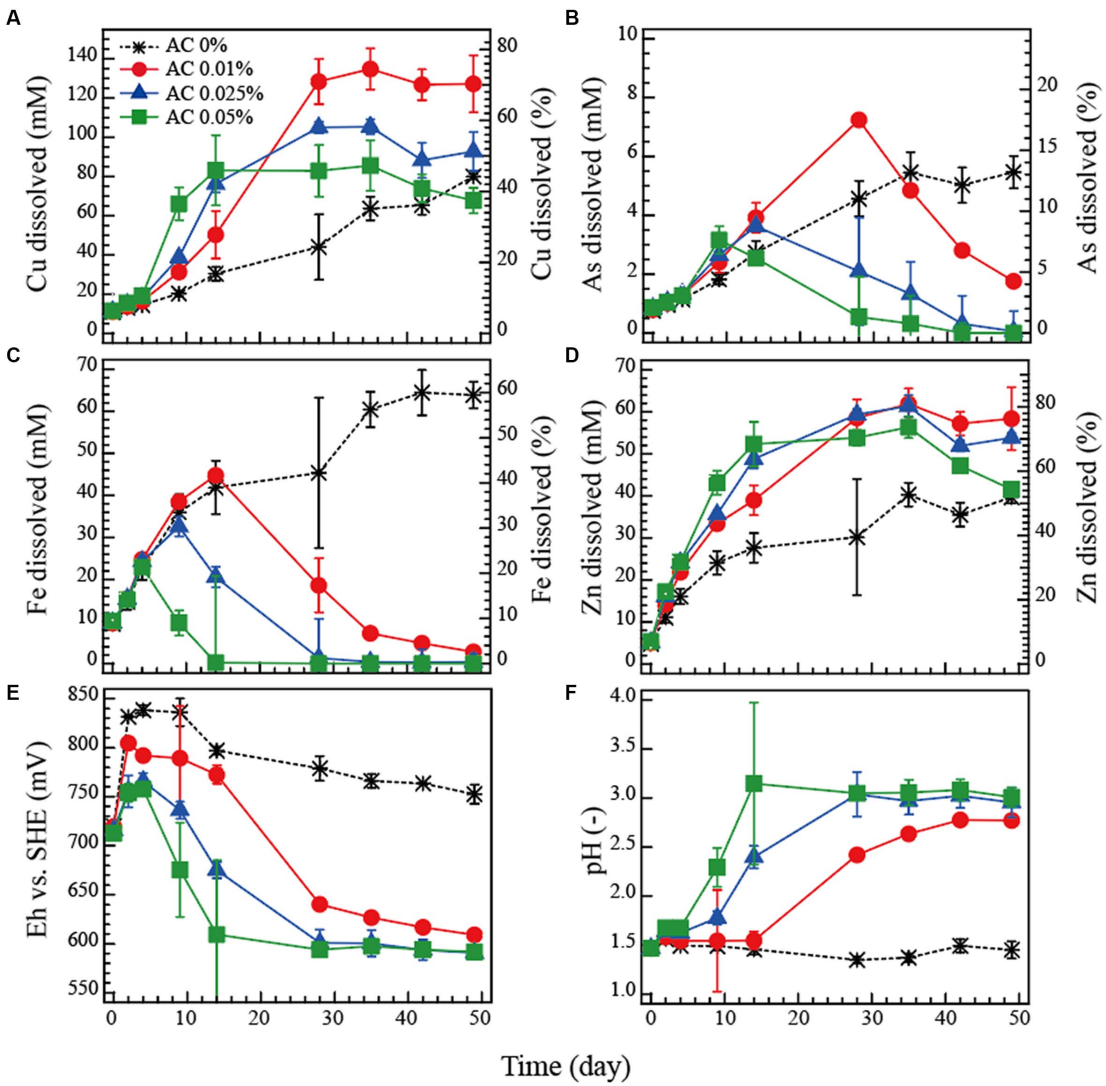
Bioleaching of tennantite concentrate in flask batch tests at 5% pulp density at 45°C. Activated carbon (AC) was added at 0, 0.01 (), 0.025 (▲) or 0.05% (■) to control the Eh level. Changes in the total soluble Cu **(A)**, As **(B)**, Fe **(C)**, Zn **(D)**, Eh vs. SHE **(E)** or pH **(F)** are shown.

The dissolution profile of metals (Cu, As, Fe and Zn) and the changes in Eh and pH during the bioleaching tests are shown in Figures 2A–F.

In the AC-free system, Eh increased rapidly to 840 mV, reflecting the high Fe-oxidising bacterial activity, and then gradually stabilised at around 780 mV (Figure 2E). pH remained stable at around 1.5 (Figure 2F). The Fe dissolution corresponded to the high Eh values in the AC-free system (Figure 2C), while Cu dissolution was the slowest of all (final Cu dissolution 45%; Figure 2A).

Compared to the AC-free system, higher AC doses (0.01, 0.025 and 0.05%) increasingly suppressed the Eh value (Figure 2E), which significantly corresponded to the Fe dissolution profile: Fe was almost completely immobilised at the time when Eh decreased to ~600 mV due to the Fe^3+^-reducing effect of AC (Figure 2C). An increase in pH due to the addition of AC may also have partially influenced the immobilisation of Fe (Figure 2F). The highest Cu dissolution (70%) was obtained in the AC-0.01% system (Figure 2A). Further increases in AC dose resulted in increasingly faster Cu dissolution in the initial phase (up to day 14), but immediately followed by a plateau which ultimately reduced the final Cu dissolution to 52 and 38% in the AC-0.025% and AC-0.05% systems, respectively (Figure 2A). Zn dissolution followed a similar trend to that of Cu, but its total percentage dissolution (Figure 2D) was generally higher than that of Cu (Figure 2A) and less influenced by Eh (Figure 2E), indicating that Zn was leached from both the sphalerite and tennantite minerals (Figure 1). The trend of As dissolution followed that of Fe (Figures 2B,C): The highest As solubilised was only 13% in the AC-free system on day 50, whereas the AC dosing effectively facilitated As immobilisation corresponding to the drop in Eh towards the end of the bioleaching period, presumably via ferric-arsenic precipitation. The concentration of total soluble Sb derived from tennantite was low throughout the bioleaching period, with final dissolution of ~0.6% (AC-free system) and ~ 0.4% (all AC systems) (data not shown).

**Figure 3:**
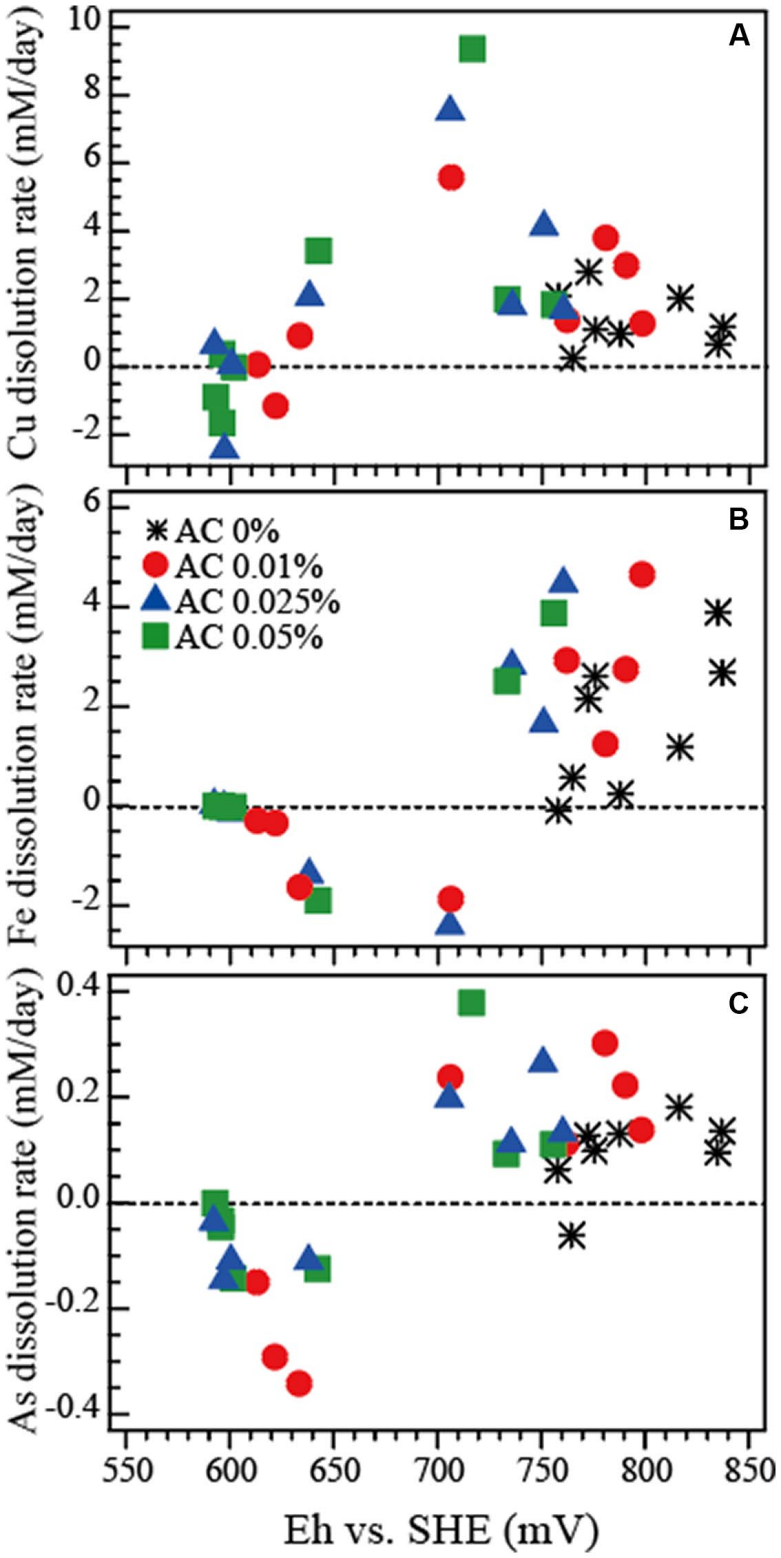
Dissolution rates of Cu **(A)**, Fe **(B)** and As **(C)** during the bioleaching of tennantite concentrate (calculated from data in Figure 2). Activated carbon (AC) was added at 0%, 0.01% (), 0.025% (▲) or 0.05% (■) to control Eh.

The dissolution rates of Cu, Fe and As (calculated from data from Figure 2) are shown in Figures 3A–C, respectively. The dissolution rate of Cu peaked at around 710–720 mV (Figure 3A), as did that of As (Figure 3C) from the tennantite mineral. The dissolution rate of Fe peaked at a higher Eh range of around 750–800 mV, reflecting the progression of pyrite leaching (Figure 3B). In order to prevent As dissolution (via As immobilisation in the form of ferric arsenate), it was found preferable to set the Eh range around 650 mV (Figures 3B,C). It was therefore suggested that maximising Cu dissolution while minimising As dissolution could be achieved by controlling the level of Eh during the bioleaching reaction.

### 3.2 Bioleaching of tennantite concentrates (pH-controlled bioreactor tests)

**Figure 4:**
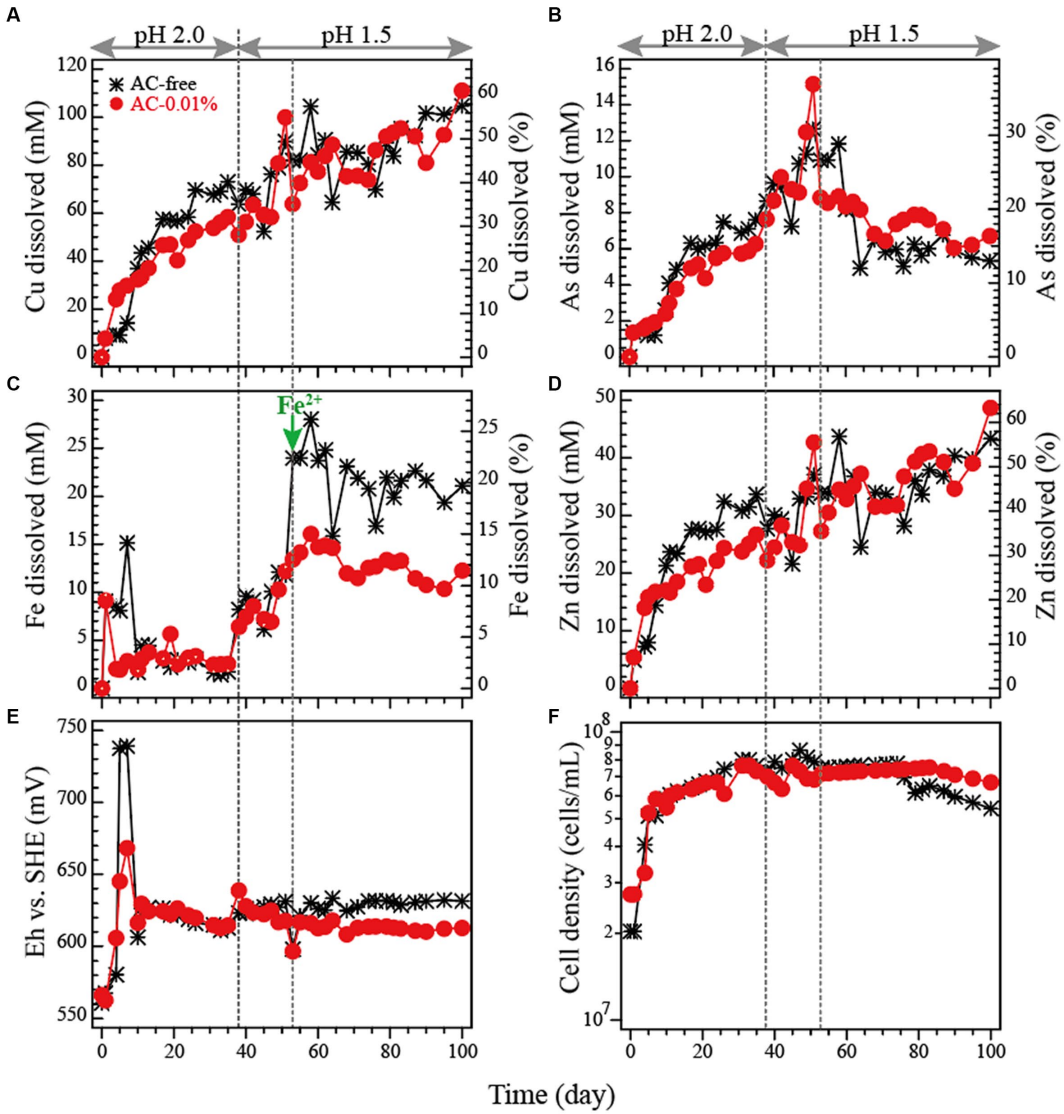
Bioleaching of tennantite concentrate in a pH-controlled bioreactor at 5% pulp density at 45°C with or without activated carbon (AC). Changes in total Cu concentration **(A)**, total As concentration **(B)**, total Fe concentration, **(D)** total Zn concentration, **(E)** Eh vs. SHE and **(F)** cell density in the AC-free system or AC-0.01% system () are shown. pH was controlled at 2.0 until day 37 and then switched to 1.5 on day 38. On day 53, either 20 mM or 10 mM of Fe^2+^ was added to the AC-free or AC-0.01% system, respectively (as indicated by a green arrow in panel **C**).

The dissolution profile of metals (Cu, As, Fe and Zn) and the changes in Eh and pH during the pH-controlled bioleaching tests are shown in Figures 4A–F. The highest Cu dissolution was obtained in the AC-0.01% system in the flask bioleaching tests. Therefore, this time either the AC-free or AC-0.01% system was compared.

Days 0–37: The initial rapid dissolution of Fe (days 0–10; Figure 4C) led to a temporal increase in Eh in both systems, although to a different extent (AC-free system ~740 mV, AC-0.01% system ~670 mV; Figure 4E). This difference was caused by the Fe^3+^-reducing (i.e., Eh-suppressing) effect of AC, as was also shown in the flask bioleaching tests (Figure 2). However, the degree of this initial Eh increase in the AC-free system this time (Figure 4E) was much smaller than that observed in the flask test (~840 mV; Figure 2F). Apart from this temporal jump in Eh, Eh generally remained low regardless of the addition of AC, gradually decreasing from 630 mV to 610 mV (Figure 4E). Since Eh was already low even in the AC-free system, the further Eh-suppressing effect of AC was no longer evident (Figure 4E). This low Eh trend was unexpected observation as active microbial cell growth was seen in both systems (Figure 4F). During days 0–37, Cu leaching progressed in both systems, although slightly more extensively in the AC-free system (Figure 4A); this difference is thought to be a remnant of a very rapid Cu dissolution that occurred around day 4 in the AC-free system, corresponding to the temporal transition of Eh to an optimal range of ~700 mV at this time (Figure 4E). After the initial jump in Fe concentration, Fe dissolution was largely suppressed during this period (Figure 4C).

Days 38–52: Since Cu leaching began to plateau slightly towards day 37, especially in the AC-free system (presumably due to the lack of oxidant Fe^3+^; Figures 4A,C), pH was reset to 1.5 on day 38 with the intention of stimulating Fe dissolution/microbial Fe^2+^ oxidation to provide more oxidant. This stimulated Fe dissolution (Figure 4C), but apparently did not result in much activation of microbial Fe^2+^ oxidation, as the increase in Eh was small (from 610 mV to 630 mV; Figure 4E). However, Cu leaching was appeared to be stimulated during this period (Figure 4A). Although the level of Eh was low and similar in both systems, a slight suppressive effect of AC on Eh was observed in the AC-0.01% system during this period (Figure 4E).

Days 53–100: As described above, the effect of AC addition was not significant in the bioreactor test because the level of microbial Fe^2+^-oxidising activity was apparently much lower than that in the flask test (Figure 2). As a result, the two systems (AC-free and AC-0.01%) were almost synchronised in all parameters by day 52 (Figure 4). In a further attempt to induce microbial Fe^2+^ oxidation (and thus increase Eh), additional Fe^2+^ was added on day 53 (20 mM to the AC-free system and 10 mM to the AC-0.01% system; Figure 4C). However, Eh was almost unchanged in the AC-free system and continued to decrease slowly in the AC-0.01% system due to the Eh suppressive effect of AC (Figure 4E). The results so far confirmed that the cause of the generally low Eh level (independent of AC) observed in the bioreactor test was not due to the lack of dissolved Fe or cell growth, but due to the lack of strong microbial Fe^2+^-oxidising activity.

**Figure 5:**
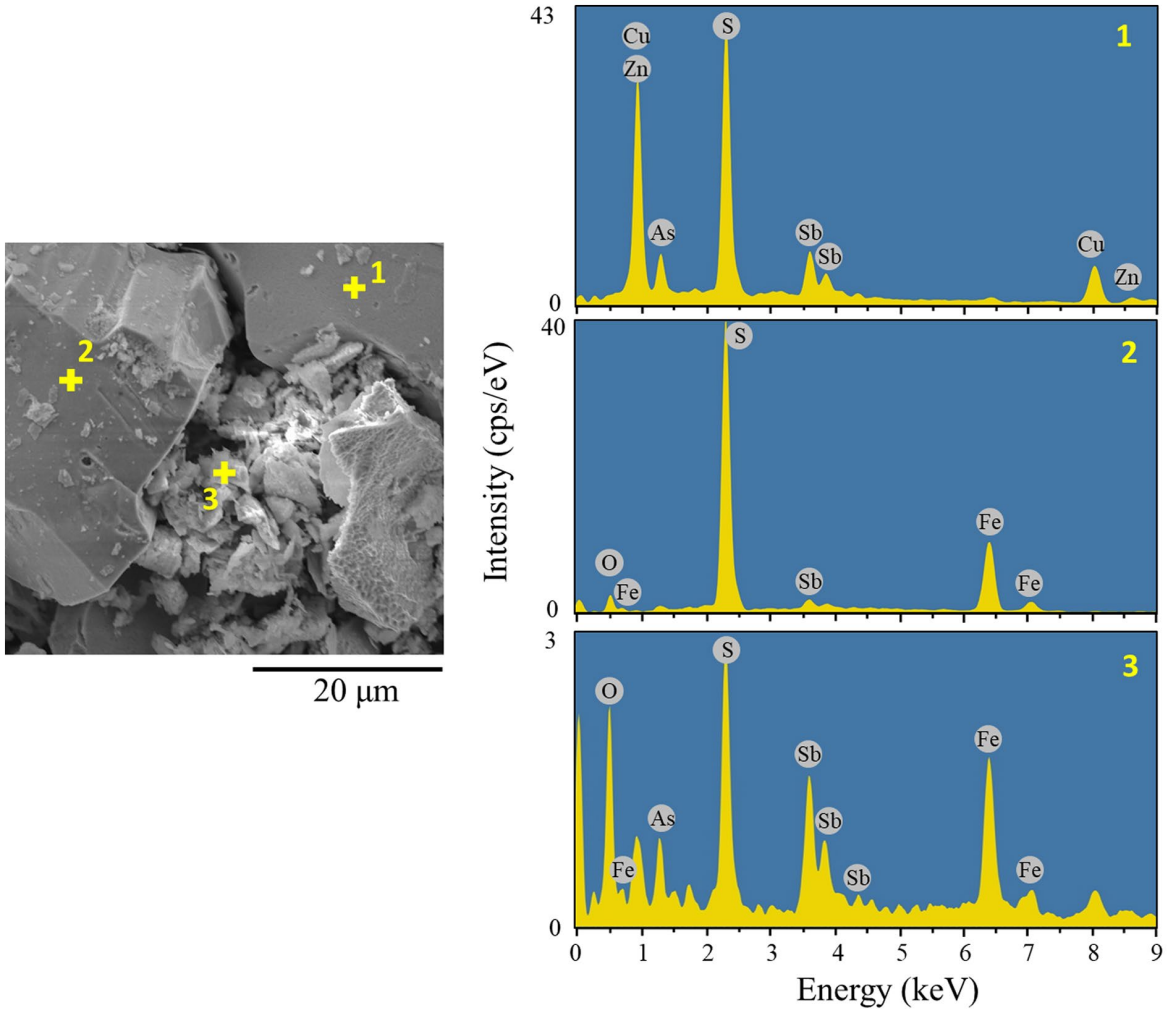
Secondary electron (SE) image and EDS analysis (Aztec Ins. X-act; 15 kV, 5 nA, point analysis mode) of the bioleached residue from the AC-0.01% bioreactor system. Point 1: tennantite mineral, Point 2: pyrite mineral, Point 3, ferric arsenate precipitate.

Overall, in this bioreactor test, the Eh level did not increase as it was originally expected and remained mostly at around 610–630 mV at all times in the AC-free system (Figure 4E). Therefore, unlike in the flask test where Eh naturally exceeded 800 mV (Figure 2E), there was no need to artificially suppress the Eh level by adding AC. The addition of AC in the bioreactor test showed some further suppression of Eh (particularly in the early and late phases; Figure 4E). According to the cell density profile (Figure 4F), although the cells grew well in both systems, the overall Fe^2+^-oxidising activity became weak for unknown reasons. As the Eh profile happened to be very similar with or without AC, the dissolution profiles of the metals were also similar: Cu leached readily to a final Cu dissolution of ~60% in both systems (Figure 4A). Fe dissolution was limited to ~20% (in the AC-free system) and ~ 11% (in the AC-0.01% system) even after the addition of Fe^2+^ on day 53 (Figure 4C). Although As originates from the mineral tennantite, its dissolution was largely suppressed compared to that of Cu. Especially after the addition of Fe^2+^ on day 53, the immobilisation of As was further facilitated, presumably as Fe^3+^-As*^V^* precipitates. Finally, only about 15% of As dissolution was found in both systems (Figure 4B). Indeed, SEM observation revealed the formation of small particles of secondary precipitates trapped between the remaining tennantite and pyrite particles (Figure 5). Elemental analysis showed that the aggregated secondary mineral particles were composed of As, Fe, O, S and Sb (Figure 5). This elemental composition suggests the possible formation of ferric arsenate (FeAsO_4_·(2 + n)H_2_O) mixed with basic ferric sulfate (MFe_x_(SO_4_)_y_(OH)_z_), as reported in our previous study (Tanaka et al., 2018). Zinc originated from the minerals tennantite and spharelite and its dissolution profile was mostly similar to that of Cu (Figure 4D).

Based on the correlation of the metal dissolution rate and Eh, the Eh range shown in this bioreactor test (Figure 4E) was mostly suboptimal in terms of the Cu dissolution rate (Figure 3A), but optimal for Fe immobilisation with As (Figures 3B,C). Our previous study (Oyama et al., 2018, 2020) showed that suppressing Fe dissolution from pyrite is advantageous to support steady and continuous leaching of Cu from the enargite concentrate (by avoiding mineral passivation by Fe minerals). This was also the case in this study as Cu leaching continued well towards the end as shown in Figure 4A.

**Figure 6:**
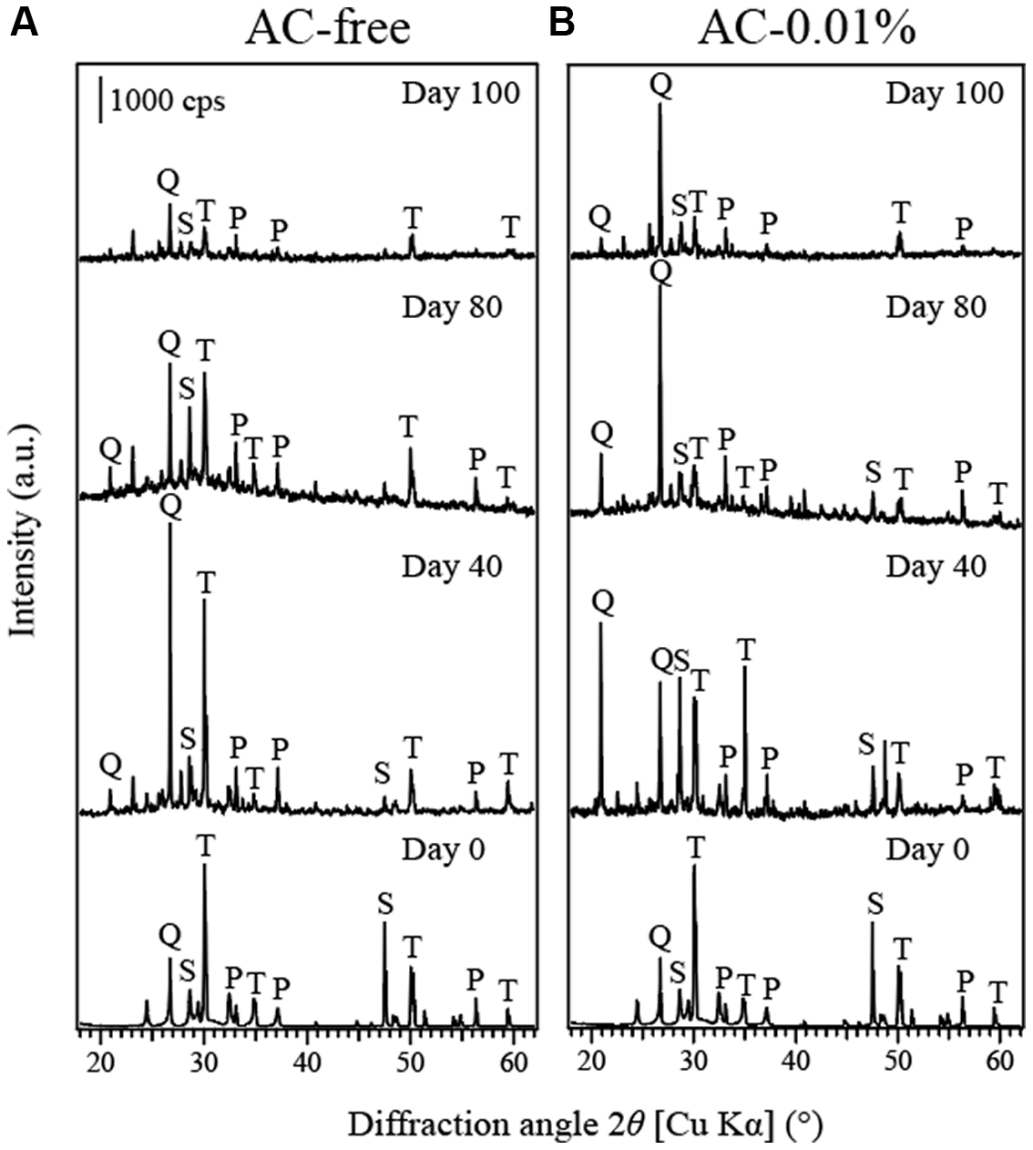
Changes in the X-ray diffraction pattern of tennantite concentrate in the AC-free **(A)** or AC-0.01% **(B)** bioreactor system on days 0, 40, 80 and 100. T: tennantite (Cu_12_As_4_S_13_; PDF No. 01–074-1027). S: sphalerite [(Zn, Fe)S; PDF No. 00–001-079], P: pyrite (FeS_2_; PDF No. 00–042-1340), Q: quartz (PDF No. 00–033-1161).

The XRD results identified the peaks of tennantite, pyrite and sphalerite in the original tennantite concentrate, of which the tennantite peaks decreased significantly as the bioleaching progressed in both the AC-free and AC-0.01% systems (Figures 6A,B, respectively). Relative to tennantite, pyrite peaks tended to persist throughout the bioleaching reaction, probably due to the low Eh conditions created during the bioreactor tests, which facilitated tennantite leaching while preventing the pyrite dissolution (Oyama et al., 2018, 2020; Okibe et al., 2022).

The mixed culture originally prepared for the first flask bioleaching test consisted of equal cell densities of six strains, including both “strong” (*Am*. *ferrooxidans*; *Acidiplasma* sp. Fv-Ap) and “weak” (*Sb*. *sibiricus*; *Sb. thermotolerans*; *Sb. thermosulfidooxidans*) Fe oxidisers together with S-oxidising *At*. *caldus* (Masaki et al., 2018). The next bioreactor tests were inoculated with the mixed culture that had undergone the flask tests. Due to the unexpectedly low Eh value observed throughout the bioreactor tests, microbial community analysis was performed on the final bioleach residue from both the flask and the bioreactor to see the transition of the microbial flora. Surprisingly, the bioleach residue from the flask was 99.9% dominated by *Sb. thermosulfidooxidans*. Consequently, the bioleach residue from the bioreactor was also dominated by this bacterium.

According to Table 1, *Sulfobacillus* spp. especially *Sb. thermosulfidooxidans* and *Sb. sibiricus* are highly resistant to Cu. In the flask bioleaching test in this study, Cu concentrations exceeded >100 mM (~6 g/L; Figure 2A) during bioleaching of 5% tenantite concentrate, which may have contributed to the convergence of the original microbial community to these *Sulfobacillus* spp. The total As concentration increased transiently up to 7.5 mM (~0.56 g/L) in the flask tests (Figure 2B) and up to 15 mM (~1.1 g/L) in the bioreactor tests (Figure 4B). Although *Sb. thermosulfidooxidans* and *Sb. sibiricus* were reported to tolerate up to 50 mM (~3.7 g/L) and 100 mM (~7.5 g/L) of As(V), respectively, the trend was reversed for As(III), with *Sb. sibiricus* being more sensitive to As(III) (Table 1). Arsenic dissolved from tenantite concentrates should originally be As(III), but is partially oxidised to As(V) by Fe^3+^ on the surface of sulfide minerals, which have a “semi-conductive” nature (Takatsugi et al., 2011; Tanaka et al., 2015). The fact that *Sb. thermosulfidooxidans* was relatively highly tolerant to both Cu and As(III) may have contributed to its proliferation as a dominant species in the microbial community.

**Table 1:** Cu and As tolerance of *Sulfobacillus* spp. compared to *Am. ferrooxidans*, *Acidiplasma* spp. and *At. caldus*.

		Element			Reference
		Cu(II)	As(V)	As(III)	
*Am. ferrooxidans*	DSM 10331^T^	9.4 mM^a^	nd	nd	Watkin et al. (2009)
		nd	nd	6.5 mM^a^	Okibe and Fukano (2019)
*Acidiplasma* spp.	Fv-Ap	nd	nd	nd	
	MBA-1, V1^T^, BH2^T^	5, 25, 10 mM^b^	nd	nd	Bulaev et al., 2017
*Sb. thermosulfidooxidans*	DSM 9293^T^	300 mM^a^	50 mM^a^	nd	Watling et al. (2008)
		nd	nd	26 mM^a^	This study
*Sb. thermotolerans*	DSM 17362^T^	25 mM^a^	50 mM^a^	nd	Watling et al. (2008)
		nd	nd	13 mM^a^	Okibe and Fukano (2019)
*Sb. sibiricus*	DSM 17363^T^	300 mM^a^	100 mM^a^	nd	Watling et al. (2008)
		nd	nd	2.6 mM^a^	Okibe and Fukano (2019)
*At. caldus*	DSM 8584^T^	24 mM^a^	nd	nd	Watkin et al. (2009)

a Highest metal concentration in which microbial growth was observed.

b MIC (minimal inhibitory concentrations).

Johnson et al. (2007) reported that the concentrate mineralogy can dictate the microbial consortia and *Sb. thermosulfidooxidans* was also found to exclusively dominate the bioleaching culture of a chalcopytite concentrate originally inoculated with a total of 12 moderately thermophilic acidophiles. Elkina et al. (2021) studied the bioleaching of a chalcopyrite/tennantite/sphalerite concentrate using a mixed culture of *At. caldus* MBC-1, *Sb. thermosulfidooxidans* SH-1 and *Acidiplasma* sp. MBA-1 at a pulp density of 2% and varying temperatures (40–60°C). The authors reported that Cu leaching improved with increasing temperature and in the presence of yeast extract, while zinc leaching was less dependent on temperature and yeast extract (Elkina et al., 2021): Cu leaching was maximised at 60°C, suggesting that *Sb. thermosulfidooxidans* plays a dominant and important role in the system. As seen in our study, Eh was microbiologically controlled (presumably by *Sb. thermosulfidooxidans*) at <700 mV under these conditions (Elkina et al., 2021). Arsenic leaching was also strongly suppressed due to the this low Eh and high temperature suitable for ferric arsenate precipitation. On the other hand, lowering the temperature increased the Eh to >800 mV (presumably by activating more “strong” Fe oxidisers), thereby reducing Cu leaching.

In the industrial bioleaching process of Cu sulfides with high As contents (especially at high pulp densities), it is likely that microbial consortia will naturally converge to leave highly Cu/As tolerant species such as *Sulfobacillus* spp. as shown in this study. In such cases, natural microbiological Eh control by these “weak” Fe oxidisers can be expected to support continuous Cu leaching as well as As immobilisation, while preventing extensive pyrite dissolution. However, as the industrial bioleaching process is continuously exposed to naturally occurring microbial consortia, it is possible that long-term microbial acclimatisation could lead to the emergence of highly metal-tolerant “strong” Fe oxidisers (thus providing a high Eh environment). If this is the case, the addition of external Eh regulators (such as AC) may help to enable sustainable Cu leaching while suppressing As dissolution, as was demonstrated in this study.

## 4 Conclusion

In the first flask bioleaching tests of tennantite concentrate, a steady increase in Eh (up to 840 mV) was observed, reflecting the activity of “strong” Fe-oxidisers included in the mixed culture used. In this case, AC dosing was effective in regulating the Eh range suitable for simultaneous Cu dissolution and As immobilisation (final dissolution ~70% Cu and ~ 4% As at AC 0.01%). The optimum Eh range was found to be ~700 mV for Cu dissolution (from tennantite), >750 mV for Fe dissolution (from pyrite), and < 650 mV for As immobilisation. Therefore, in order to maximise Cu dissolution and As immobilisation, it was found preferable to set the Eh range around 650–700 mV during bioleaching. The second bioreactor tests used the mixed culture of the same origin, but had heen subcultured a few generations further on tennantite concentrate. The Eh level remained unexpectedly low (~630 mV) for most of the leaching period, regardless of the AC dosage. It was later found that the bioreactors were almost exclusively dominated by *Sb. thermosulfidooxidans* (a “weak” Fe oxidiser with high Cu/As tolerance). In this case, there was no need to artificially suppress the Eh level by AC dosing and Cu leached readily to a final Cu dissolution of ~60% while As dissolution was suppressed to ~15%. The highlights of this study suggest that, depending on the microbial community that develops at the processing site, Eh control can be achieved either naturally through the activity of “weak” Fe-oxidisers as the predominant survivors under high Cu/As stress, or artificially by the addition of an Eh regulator such as a carbon catalyst.

## Data availability statement

The original contributions presented in the study are included in the article/supplementary material, further inquiries can be directed to the corresponding author.

## Author contributions

SK: Investigation, Writing – original draft. KH: Investigation, Writing – original draft. IP: Investigation. NO: Conceptualization, Resources, Supervision, Funding acquisition, Validation, Methodology, Project administration, Writing-review & Editing.
